# Development of Monoclonal Antibody against PirB and Establishment of a Colloidal Gold Immunochromatographic Assay for the Rapid Detection of AHPND-Causing *Vibrio*

**DOI:** 10.3390/ani14111600

**Published:** 2024-05-29

**Authors:** Xuan Dong, Jingmei Xie, Liying Wang, Xuan Li, Haoyu Lou, Guohao Wang, Jie Huang

**Affiliations:** 1 State Key Laboratory of Mariculture Biobreeding and Sustainable Goods, Yellow Sea Fisheries Research Institute, Chinese Academy of Fishery Sciences, Laboratory for Marine Fisheries Science and Food Production Processes, Laoshan Laboratory, Key Laboratory of Maricultural Organism Disease Control, Ministry of Agriculture and Rural Affairs, Qingdao Key Laboratory of Mariculture Epidemiology and Biosecurity, Qingdao 266071, China; 15147315297@163.com (J.X.); wly1810060249@163.com (L.W.); lixuan00213@163.com (X.L.); 18790558853@163.com (H.L.); wgh15866019360@163.com (G.W.); huangjie@ysfri.ac.cn (J.H.); 2 Jiangsu Shufeng Aquatic Seed Industry Co., Ltd., Gaoyou 255654, China

**Keywords:** AHPND, *Vibrio*, PirB, monoclonal antibody, colloidal gold immunochromatographic assay

## Abstract

**Simple Summary:**

This research aims to develop rapid and user-friendly diagnostic approaches for the detection of acute hepatopancreatic necrosis disease (AHPND), a lethal disease threatening the shrimp industry worldwide. We have developed a special antibody to target a virulence protein in the bacteria that cause this disease. By using this antibody, we have created a rapid and accurate test using gold particles to detect these harmful bacteria. The test has shown promising results, accurately distinguishing between disease-causing and harmless bacteria. This innovative method could revolutionize the early detection and management of AHPND in shrimp farms, leading to healthier shrimp production and improved disease control. Ultimately, this advancement has the potential to benefit the aquaculture industry by safeguarding shrimp populations and ensuring sustainable farming practices.

**Abstract:**

Acute hepatopancreatic necrosis disease (AHPND) poses a significant threat to shrimp aquaculture worldwide, necessitating the accurate and rapid detection of the pathogens. However, the increasing number of *Vibrio* species that cause the disease makes diagnosis and control more difficult. This study focuses on developing a monoclonal antibody against the *Photorhabdus* insect-related (Pir) toxin B (PirB), a pivotal virulence factor in AHPND-causing *Vibrio*, and establishing a colloidal gold immunochromatographic assay for the enhanced early diagnosis and monitoring of AHPND. Monoclonal antibodies targeting PirB were developed and utilized in the preparation of colloidal-gold-labeled antibodies for the immunochromatographic assay. The specificity and sensitivity of the assay were evaluated through various tests, including antibody subclass detection, affinity detection, and optimal labeling efficiency assessment. The developed PirB immunochromatographic test strips exhibited a good specificity, as demonstrated by the positive detection of AHPND-causing *Vibrio* and negative results for non-AHPND-causing *Vibrio*. The study highlights the potential of the developed monoclonal antibody and immunochromatographic assay for the effective detection of AHPND-causing *Vibrio*. Further optimization is needed to enhance the sensitivity of the test strips for improved practical applications in disease prevention and control in shrimp aquaculture.

## 1. Introduction

Acute hepatopancreatic necrosis disease (AHPND) is a bacterial disease that significantly impacts shrimp aquaculture and is listed by the World Organisation for Animal Health (WOAH) as a notifiable aquatic animal disease [1]. Initially, AHPND was attributed to *Vibrio parahaemolyticus* strains carrying the pVA1 plasmid, which harbors genes responsible for producing counterparts of the *Photorhabdus* insect-related (Pir) toxins, namely, PirA and PirB [1,2,3]. However, recent studies have revealed a broader range of pathogens causing AHPND. *V. owensii* carrying the pVA1 plasmid was isolated from AHPND-infected shrimp [4,5]. Additionally, strains of *V. campbellii* causing AHPND and carrying the pVA1 plasmid were identified [6,7,8]. Furthermore, it was found that *V. harveyi* carrying the pVA1 plasmid could also induce AHPND [9]. Research has also indicated that the type IV secretion system (T4SS) can mediate the conjugative transfer of the pVA1 plasmid carrying *pirAB* genes, increasing pathogen diversity [10]. Therefore, PirAB is identified as the main target protein of AHPND-causing *Vibrio* [2,11].

Clinical signs of AHPND-infected shrimp are challenging to distinguish from other pathogen-induced signs, making an accurate diagnosis solely based on clinical observations difficult. Laboratory testing is essential for an accurate diagnosis. The rapid and accurate identification of the pathogen plays a crucial role in AHPND prevention and control [12,13,14]. While PCR detection methods offer a high sensitivity, they require skilled operators and specialized equipment, posing limitations. Notably, the partial absence of the *pirA* and/or *pirB* genes or the presence of the full-length genes without the expression of virulence proteins can lead to false positives during detection [6,15,16,17]. Therefore, there is an urgent need for a detection method targeting virulence proteins. A small number of studies have developed antibodies and immunological methods targeting PirA or PirB [15,18,19,20,21,22], primarily focusing on AHPND-causing *V. parahaemolyticus* strains without a comprehensive evaluation of other AHPND-causing *Vibrio* species.

The colloidal gold immunochromatographic assay is a rapid detection method that utilizes the specific binding of antigens and antibodies to provide a quick analysis of detection products [23,24,25,26]. This method has found widespread application in animal pathogen detection, medical diagnostics, and food safety testing [23,24,25,26]. This study aims to develop monoclonal antibodies against the virulence protein PirB of AHPND and establish a colloidal gold immunochromatographic assay for the rapid detection of AHPND-causing pathogens. This advancement will complement existing detection methods for AHPND-causing *Vibrio*, providing technical support for the effective prevention and control of AHPND.

## 2. Materials and Methods

### 2.1. Bacterial Strains and Plasmid

All strains and the plasmid used in this study are depicted in Table 1. The bacterial strains were retrieved from a −80 °C ultra-low temperature freezer containing glycerol stocks. After thawing at room temperature until completely dissolved, 10 µL of the bacterial suspension was inoculated onto suitable solid culture media. Using a new tip, we performed streaking on 2216E or LB solid media, followed by incubation at 28 °C or 37 °C for 12 h. Single colonies were then picked and inoculated into sterile 10 mL liquid 2216E or LB media in a shaker incubator at 28 °C or 37 °C with a speed of 180 rpm for 12 h. For cultures requiring antibiotics, extended incubation was necessary before subsequent experiments.

### 2.2. Expression, Purification, and Identification of Recombinant PirB Protein

The synthetic modified *pirB* gene sequence was designed based on the virulence gene characteristics of AHPND-causing *Vibrio*. *Eco*RI and *Xho*I restriction sites were inserted upstream and downstream. The target fragment of modified *pirB* with enzyme restriction site sequences was 1326 bp. The gene was cloned into the linearized pET-30a(+) vector to obtain a recombinant plasmid pET-30a(+)-*pirB*. *E. coli* BL21 competent cells stored at −80 °C were thawed slowly on ice. The plasmid was mixed with the cells, heat-shocked at 42 °C for 90 s, and then incubated at 37 °C for 45 min. Positive clones were identified by transferring single colonies to LB with Ampicillin, followed by plasmid extraction and gel electrophoresis. The recombinant plasmid was induced for expression in BL21 cells using isopropyl β-D-1-thiogalactopyranoside (IPTG), followed by cell lysis and SDS-PAGE analysis. For the bulk expression of PirB protein, the bacterial cells were resuspended in 20–30 mL of 10 mM Tris-HCl solution (pH 8.0) and disrupted using ultrasonication (500 W, 180 cycles, 5 s on, 5 s off). Protein purification was carried out using a His-tag protein purification kit from Solarbio (Beijing, China).

### 2.3. Mouse Immunization

Recombinant PirB protein was chosen as the immunogen for mouse immunization. Four randomly selected specified pathogen-free (SPF)-grade female BALB/c mice labeled with #1, #2, #3, and #4 received subcutaneous injections of PirB recombinant protein at 60 μg per mouse for the primary immunization. Mice were subjected to the first booster immunization at a 14-day interval, with subcutaneous injection of immunogen containing recombinant protein at 30 μg per mouse; after another 14-day interval, mice received the second booster immunization with subcutaneous injection of immunogen containing recombinant protein at 30 μg per mouse; followed by a further 14-day interval, mice underwent the third booster immunization with subcutaneous injection of immunogen containing recombinant protein at 30 μg per mouse. Post-boost immunization, the antibody titer was assessed. Serum samples were tested by coating with PirB solution at 2 μg/mL overnight at 4 °C, followed by blocking with phosphate-buffered saline (PBS) containing 2% skim milk at 37 °C for 2 h. Serum dilutions starting from 1:200 were tested in 2-fold gradients, with PBS as the blank control and negative serum at a 1:200 dilution as the negative control.

### 2.4. Hybridoma Cell Fusion and Screening

Mouse #1 was selected for cell fusion based on the antibody titer results. Mouse spleen cells and SP2/0 cells were fused using the polyethylene glycol (PEG) method [27,28]. The fused cells were then cultured in a semi-solid medium containing hypoxanthine, aminopterin, and thymidine (HAT) for selection. The resulting cells were then subjected to PEG treatment, followed by selection and culturing in 30 cell culture dishes. Single-cell clones were picked from 96-well plates for further analysis.

For screening, wells of a new 96-well plate were coated with the PirB solution in coating buffer overnight, followed by washing and blocking steps. Primary antibody (cell culture supernatant), negative control (SP2/0 culture supernatant), blank control (PBS), and positive control (PirB-positive serum) were added. After incubation and washing, goat anti-mouse IgG/horseradish peroxidase (HRP) was applied. Substrate was added for color development, and absorbance was measured. Positive cell clones were further screened using ELISA with PirB and His tag coating, and positive hybridoma cell clones were selected. The selected positive cell clones underwent subclass identification.

### 2.5. Detection of Optimal Antibody Labeling Efficiency for Immune Colloidal Gold Test Strips

To determine the optimal antibody labeling efficiency for the immune colloidal gold test strips, we selected antibodies based on antibody pairing results. Affinity testing was conducted on purified antibodies, followed by testing the labeling efficiency using HRP-conjugated antibodies diluted in PBS with Tween 20 (PBST) with 2% skim milk. Subsequently, sandwich ELISA pairing tests were performed between the antibodies on a 96-well microplate coated with the capture antibody. Initially, for antibody coating, the capture antibody was diluted in coating buffer solution (CBS) to 5 µg/mL and added to the microplate (100 µL per well) for overnight incubation at 4 °C. Subsequent blocking involved washing with PBST, air-drying the plate, and blocking with 2% skim milk in 1× PBS at 37 °C for 2 h (200 µL per well). Sandwiching involved washing with PBST, air-drying, adding the sandwich recombinant PirB samples at a specific dilution ratio (100 µL per well), and incubating at 37 °C for 1 h. Following incubation, the detection antibody was added, washed, and developed using the 3,3′,5,5′-Tetramethylbenzidine (TMB) substrate. The reaction was stopped, and absorbance values at 450 nm and 630 nm were measured to draw experimental results.

### 2.6. Preparation of Immune Colloidal Gold Test Strips for PirB

Test strips were prepared by Beijing Protein Innovation Co., Ltd. (Beijing, China). The preparation of immune colloidal gold test strips involved synthesizing colloidal gold solution using the sodium citrate reduction method with slight modifications [25]. The process included adding 100 mL 0.03% (*w*/*w*) chloroauroic acid solution, stirring, and heating to boiling on the magnetic stirrer. Quickly add 5 mL 1% (*w*/*w*) sodium citrate solution, and continue to heat and boil for 5–10 min. When the color of the solution gradually changes from dark blue to a bright orange-red color, stop boiling and return to the original volume of 100 mL. Sample pads were made from glass fiber with buffer and stored at room temperature. The optimal detection antibody was combined with colloidal gold conjugate and fixed on polyester fiber. The conjugate pad dried at 37 °C for 5 h. The capture antibody and goat anti-mouse specific antibody were respectively micro-sprayed onto a nitrocellulose membrane at a position that would become the test line and control line. Test strips were assembled by layering a sample pad, conjugate pad, absorbent pad, and nitrocellulose membrane with test and control lines on a backing card.

### 2.7. Animal Infection Experiment

To obtain specific animal tissue samples post-infection with AHPND-causing *Vibrio*, immersion challenge experiments of *P. vannamei* were conducted using *Vc*LMB29, *Vc*LMB29-pVPGX1, and *Vp*2S01 [3]. The *Vp*2S01-infected group served as the positive control, the *Vc*LMB29-pVPGX1-infected group as the experimental group, and the *Vc*LMB29-infected group as the negative control, with sterile seawater used as the blank control. The main steps included bacterial strains were thawed from a −80 °C freezer, inoculated in 2216E liquid medium, and incubated at 28 °C for 12 h until reaching an OD_600_ of 0.50. The bacterial suspension was centrifuged, resuspended in sterile seawater, and mixed to create an infection solution with a concentration of 1 × 10^8^ CFU/mL. Shrimp were immersed in the solution for 15 min, followed by continuous immersion in a 30 L tank with a concentration of 1 × 10^6^ CFU/mL. Observations were made every 6 h for 48 h post-infection, including feeding, water quality monitoring, and shrimp behavior assessment. Tissue samples were collected for the detection of immunochromatographic test strips and molecular detection of the AP1 and *pirB* gene, and precautions were taken to prevent the spread of experimental strains post-experiment.

### 2.8. Detection of Virulence Genes in Experimental Infection Samples and Bacteria Samples

Hepatopancreas samples of moribund shrimp post-infection were promptly collected at each observation time point for mixed DNA extraction, followed by virulence gene detection. Shrimp DNA extraction was performed using the Marine Animal Tissue Genomic DNA Extraction Kit from TianGen Biotech (Beijing, China), with subsequent DNA concentration measurement. Then, 1 µL of DNA was used as the PCR template, and the remainder was stored at −20 °C.

The bacterial strains utilized in this study included AHPND-causing *Vibrio* strains of *Vp*2S01::*cat*, *Vp*2S01, *Vc*3S01, 20230718001-5, and X170302, and non-AHPND-causing strains of *Vc*LMB29, 20211214002-3, 20220331001-6, 20170907027, and 20230524001 (Table 1). After activation and cultivation, with OD_600_ reaching approximately 0.50, bacterial genomic DNA was extracted using a bacterial genome DNA extraction kit to serve as the PCR template.

Following established methods, virulence gene detection was conducted on experimental samples using primers AP1F/R and VpPirB-392F/R [11,29].

### 2.9. SDS-PAGE and Western Blot Analysis

For SDS-PAGE analysis, bacterial strains including *Vp*2S01, *Vp*2S01::*cat*, *Vc*3S01, X170302, *Vc*LMB29, 20230718001-5, 20211214002-3, 20220331001-6, 20170907027, and 20230524001 were utilized (Table 1). The procedure involved inoculating bacterial cultures, sonicating, and running samples on an 8% protein gel for band visualization. In western blot analysis, proteins were transferred onto a polyvinylidene fluoride (PVDF) membrane, blocked, probed with primary antibody PirB, incubated with HRP-conjugated secondary antibody, and developed for imaging following the same electrophoresis steps as SDS-PAGE.

### 2.10. Detection of AHPND-Causing Vibrio and Tissue Samples Using Immunochromatographic Strip

For immunochromatographic strip’s analytical specificity analysis, bacterial strains, including *Vp*2S01, *Vp*2S01::*cat*, *Vc*3S01, X170302, *Vc*LMB29, 20230718001-5, 20211214002-3, 20220331001-6, 20170907027, and 20230524001, were utilized (Table 1). The process included culturing bacterial strains at 28 °C or 37 °C overnight to an OD_600_ = 0.50, sonicating the cultures (crushing 2S, interval 3S, crushing time 10–15 min), diluting samples (10-fold dilution), and analyzing results after 15 min of strip application in an Eppendorf tube. To evaluate analytical sensitivity, PirB protein (0.4 mg/mL) was diluted in PBST to concentrations of 400 ng/mL, 40 ng/mL, and 4 ng/mL, with PBST as the blank control. Subsequently, 300 µL of each PirB test solution was added to separate 1.5 mL Eppendorf tubes, followed by the insertion of the test strips for reaction. After an incubation period of 10–20 min, the results were observed. In order to detect virulence protein in infected tissue samples, 30 mg of infected shrimp hepatopancreas was added with 300 µL 1× sample dilution, homogenized, and centrifuged for 1 min at 12,000× *g*. The test strip was inserted into the homogenate. The resulting strips were observed after 15–20 min.

## 3. Results

### 3.1. PirB Protein Purification and Identification Results

**Figure 1:**
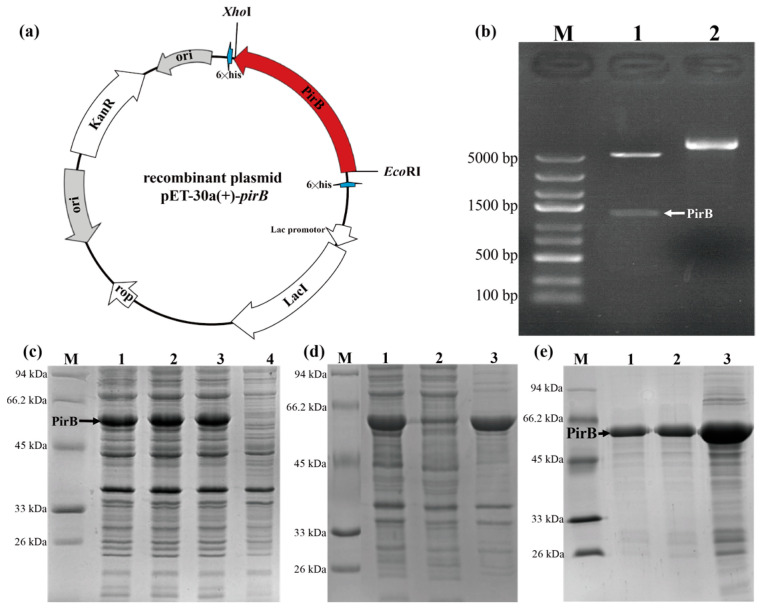
Verification and purification of recombinant protein PirB. (**a**) Recombinant plasmid map of pET-30a(+)-*pirB*. (**b**) Identification results of PirB recombinant plasmid. M: DNA Marker DL 5000; 1: Plasmid digested by *Eco*RI and *Xho*I; 2: Plasmid DNA. (**c**) The small-amount expression gel map of the PirB protein. M: Marker; 1–3: IPTG-induced recombinant BL21 expression bacteria; 4: Recombinant BL21 expression bacteria. (**d**) Bulk expression gel map of PirB protein. M: Marker; 1: Ultrasonically crushed whole bacteria; 2: Ultrasonic crushing with supernatant; 3: Sedimentation after ultrasonic crushing. (**e**) PirB protein mass purification and expression gel map. M: Marker; 1: 10-fold dilution of purified protein; 2: 5-fold dilution of purified protein; 3: Original sample of purified protein.

Gel electrophoresis of the PirB recombinant plasmid positive clone post double enzyme digestion confirmed the successful construction of the PirB recombinant plasmid, displaying a target band around 1326 bp (Figure 1a,b). The induction of the PirB recombinant plasmid in BL21 cells resulted in the successful expression of the PirB protein. The SDS-PAGE analysis revealed a PirB protein band around 50 kDa, indicating effective protein expression (Figure 1c). The large-scale expression showed the presence of PirB target protein bands around 50 kDa in the lysate and pellet but not in the supernatant, suggesting a protein presence in the inclusion bodies (Figure 1d). The purification and subsequent SDS-PAGE analysis confirmed the presence of purified PirB protein bands around 50 kDa (Figure 1e). The ELISA analysis of serum samples from immunized BALB/c mouse #1 revealed the highest OD_450_ at each serum dilution (Table 2), leading to the selection of mouse #1 for cell fusion experiments. The screening and subcloning of positive hybridoma cells resulted in the selection of positive hybridoma cell clones based on ELISA measurements.

### 3.2. Detection Results of Virulence Genes and Proteins in Experimental Bacteria Samples

The results of PCR detection showed that positive AP1 and *pirB* genes were detected in *Vp*2S01, *Vc*3S01, X170302, and 20230718001-5, while AP1 and *pirB* genes were not detected in *Vc*LMB29, 20211214002-3, 20220331001-6, 20170907027, and 20230524001, as illustrated in Figure S1.

**Figure 2:**
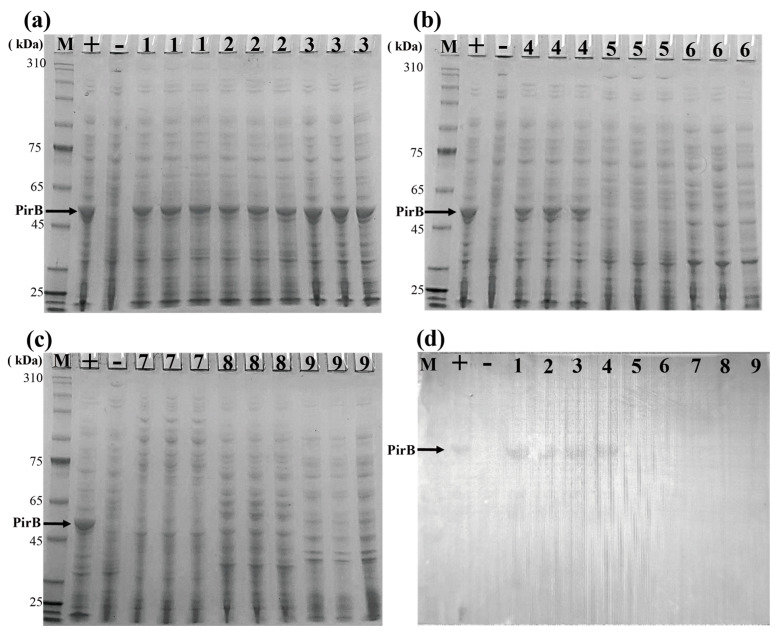
SDS-PAGE and western blot results of PirB protein of strains. (**a**–**c**) SDS-PAGE results; (**d**) western blot results. M: Marker; +: Positive control (*Vp*2S01::*cat*); −: Negative control (*Vc*LMB29); 1: *Vp*2S01; 2: *Vc*3S01; 3: X170302; 4: 20230718001-5; 5: *Vc*LMB29; 6: 20211214002-3; 7: 20220331001-6; 8: 20170907027; 9: 20230524001.

Selected bacterial strains *Vp*2S01, *Vc*3S01, X170302, *Vc*LMB29, 20230718001-5, 20211214002-3, 20220331001-6, 20170907027, and 20230524001 underwent an SDS-PAGE and corresponding western blot analysis for PirB protein detection. The findings revealed that the SDS-PAGE and western blot results for *Vp*2S01, *Vc*3S01, X170302, and 20230718001-5 exhibited PirB protein bands and immunoblots, while *Vc*LMB29, 20211214002-3, 20220331001-6, 20170907027, and 20230524001 did not show PirB protein bands or immunoblots, as depicted in Figure 2a–d. Therefore, the specificity of the PirB monoclonal antibody was demonstrated as it reacted exclusively with AHPND-causing *Vibrio* strains *Vp*2S01, *Vc*3S01, X170302, and 20230718001-5, indicating its high specificity.

### 3.3. Detection Results of Optimal Antibody Labeling for Immunochromatographic Test Strips

**Figure 3:**
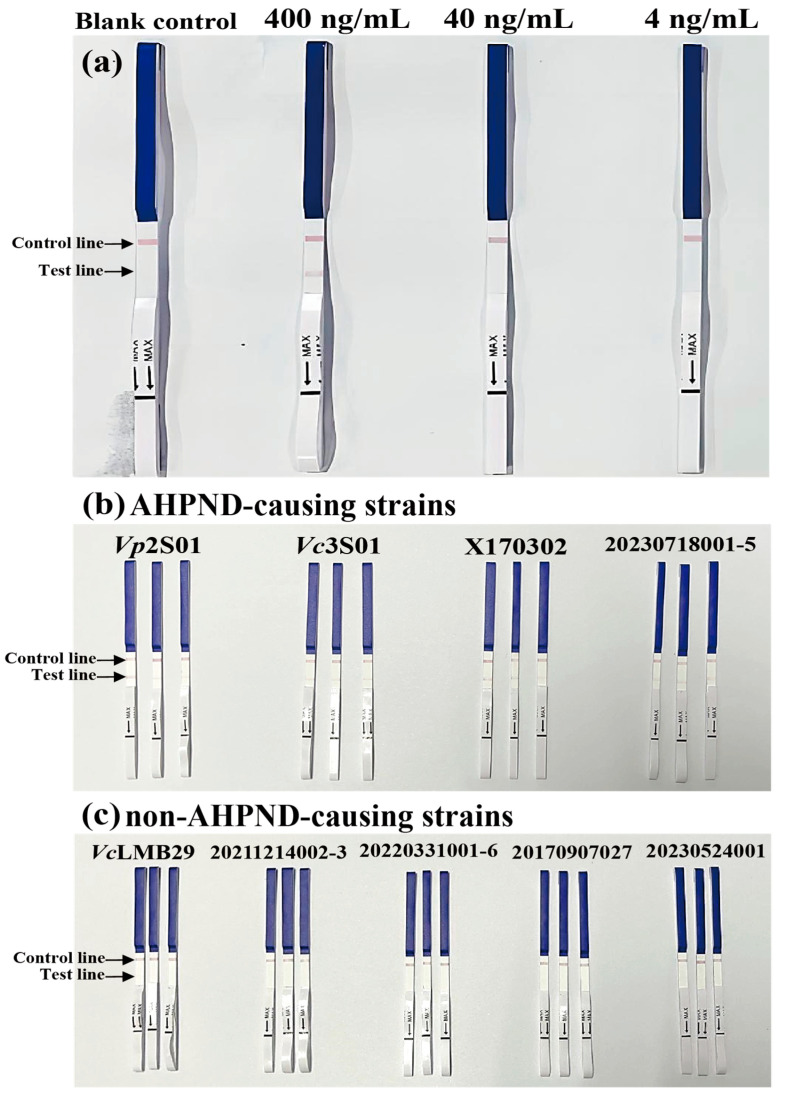
Verification of sensitivity and specificity of immunochromatographic test strips. (**a**) Analytical sensitivity results. (**b**,**c**) Analytical specificity results.

To evaluate sensitivity, we diluted PirB protein in PBST to concentrations of 400 ng/mL, 40 ng/mL, and 4 ng/mL, with PBST as the blank control. The reaction bands with 400 ng/mL protein are relatively clear, while the reaction with 40 ng/mL protein is weaker, visible to the naked eye, but cannot be distinguished from Figure 3a. The blank (PBST) reaction shows no nonspecific response (Figure 3a). The specificity analysis of immunochromatographic gold test strip demonstrated positive outcomes for AHPND-causing *Vibrio* strains *Vp*2S01, *Vc*3S01, X170302, and 20230718001-5 (Figure 3b), with negative results for non-AHPND-causing strains (Figure 3c).

### 3.4. Detection Results of Virulence Genes of Samples from the Experimentally Infected Shrimp

**Figure 4:**
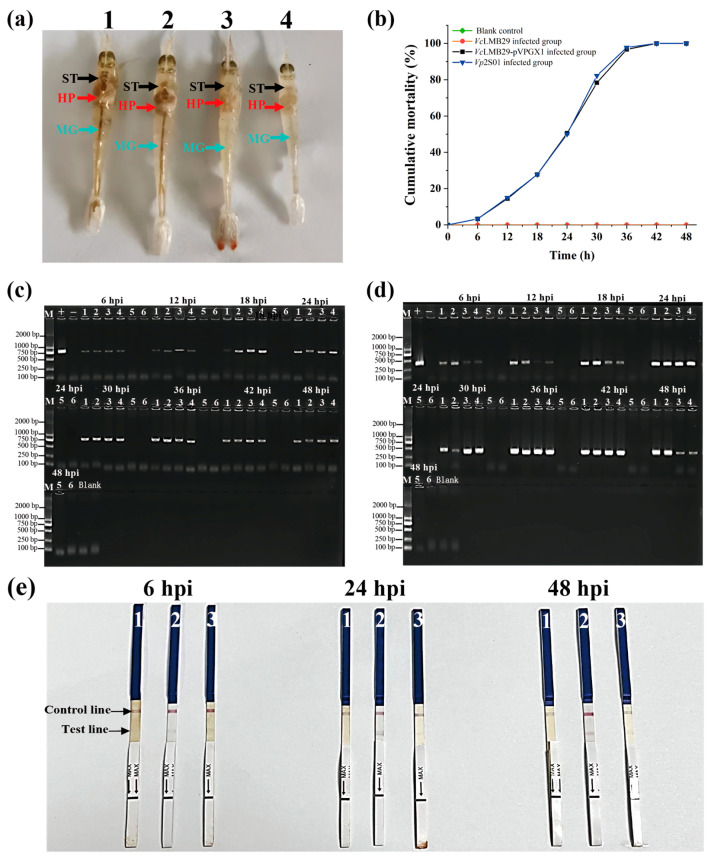
Virulence gene test results of challenge test samples. (**a**) Clinical signs of shrimp infected with AHPND-causing *Vibrio*. 1: Shrimp in the blank control group; 2: Shrimp in the *Vc*LMB29 infected group; 3: Shrimp in the *Vc*LMB29-pVPGX1 infected group; 4: Shrimp in the *Vp*2S01 infected group. ST means stomach, HP means hepatopancreas, and MG means midgut. (**b**) Cumulative mortality of shrimp infected with *Vc*LMB29, *Vc*LMB29-pVPGX1, and *Vp*2S01. (**c**) PCR results for AP1 in shrimp. hpi: Hours post infection; M: Marker; +: Positive control (*Vp*2S01::*cat*); −: Negative control (*Vc*LMB29); 1–2: *Vp*2S01 infection group; 3–4: *Vc*LMB29-pVPGX1 infection group; 5: *Vc*LMB29 infection group; 6: Blank control group. (**d**) PCR results for *pirB* in shrimp. hpi: Hours post infection; M: Marker; +: Positive control (*Vp*2S01::*cat*); −: Negative control (*Vc*LMB29); 1–2: *Vp*2S01 infection group; 3–4: *Vc*LMB29-pVPGX1 infection group; 5: *Vc*LMB29 infection group; 6: Blank control group. (**e**) Detection of PirB protein in the hepatopancreas of shrimp at different infection times by immunochromatographic strip. 1: *Vc*LMB29; 2: *Vc*LMB29-pVPGX1; 3: *Vp*2S01; hpi: Hours post infection.

A challenge test with *P. vannamei* infected with *Vc*LMB29-pVPGX1 and *Vp*2S01 strains resulted in typical AHPND clinical signs (Figure 4a), leading to a cumulative mortality rate of 100% within 48 h (Figure 4b). The PCR analysis confirmed the presence of the AP1 and *pirB* gene in shrimp infected with *Vc*LMB29-pVPGX1 and *Vp*2S01 strains, while the control group showed no detection of the AP1 and *pirB* gene (Figure 4c,d). The immunochromatographic gold test strip analysis demonstrated positive outcomes for *Vp*2S01-infected shrimp samples and *Vc*LMB29-pVPGX1-infected shrimp samples at 6 hpi, 24 hpi, and 48 hpi, with negative results for *Vc*LMB29-infected shrimp samples (Figure 4e).

## 4. Discussion

This study aimed to develop rapid and user-friendly diagnostic methods for AHPND, a severe condition that impacts shrimp health and the sustainability of aquaculture [1]. By targeting the PirB protein, a pivotal virulence factor in AHPND-causing *Vibrio*, we aim to bolster early detection and control measures to mitigate the impact of this disease.

The successful expression of the PirB protein laid a solid foundation for subsequent antibody development. Monoclonal antibodies play a pivotal role in the precise detection of pathogens due to their high affinity and specificity. Through a series of immunizations, screening, and hybridoma cell fusion techniques, we successfully obtained hybridoma cell lines capable of secreting monoclonal antibodies against PirB. Compared to polyclonal antibodies [21,22], monoclonal antibodies have a better specificity and a more stable production capacity because of hybridoma cell lines. This achievement underscores the significance of monoclonal antibodies in the accurate detection of AHPND-causing *Vibrio*.

The establishment of the colloidal gold immunochromatographic assay signifies a notable technological advancement in disease detection within aquaculture settings. By combining colloidal gold nanoparticles with specific monoclonal antibodies against PirB, we engineered immunochromatographic test strips with a high analytical sensitivity and specificity. Compared to the established immunochromatographic strip test using the monoclonal antibody of PirB for AHPND-causing *V. parahaemolyticus* (6.25 μg/mL of the toxin) [20], our method exhibits a higher detection sensitivity. The test strips exhibited a low detection limit of 400 ng/mL of PirB protein, rendering them a valuable tool for the early and rapid detection of AHPND-causing *Vibrio*. The sensitivity of the test strip was lower than that of molecular detection methods with amplification. Previous papers reported that the limited sensitivity of the strip was about 1000 times less than PCR [20]. However, the application scenario of the test strip is different. The test strip can be operated quickly at the shrimp pond without the need for equipment. The main purpose of this operation is to quickly diagnose diseased cases and determine whether the pathogen is the AHPND-causing *Vibrio* rather than carry out the detection in the lab for screening pathogens in the early stage of a disease or an unknown disease state like PCR. In addition, the test strip we established has been able to achieve the specific detection of samples 6 h after infection (Table S1 and Figure S2). On the other hand, PCR, loop-mediated isothermal amplification (LAMP), recombinase polymerase amplification (RPA), and CRISPR/Cas12a-based rapid molecular detection methods offer high sensitivities [12,13,14,29,30,31,32,33]. However, the virulence of AHPND-causing *Vibrio* relies on the expression of PirAB proteins rather than the copy number of *pirAB* genes [34]. In addition, the partial absence of the *pirA* and/or *pirB* genes or the presence of the full-length genes without the expression of virulence proteins can lead to the PCR results showing as positive, yet with the PirA/PirB protein remaining unexpressed [6,15,16,17]. Some bacterial strains carrying both *pirA* and *pirB* genes do not cause AHPND [17]. This scenario can potentially result in false positives in the PCR detection. For the above reasons, the comparison of the strips with molecular assays and the analysis of diagnostic specificity and sensitivity were not carried out in this study. We will use more experiments and data to confirm this information in our future work. The prompt and accurate detection of AHPND-causing *Vibrio* is imperative for timely intervention and effective disease control in shrimp aquaculture. The immunochromatographic assay offers a user-friendly, cost-effective, and efficient method for the on-site screening of AHPND-causing *Vibrio*. Detecting the presence of AHPND-causing *Vibrio* at early stages empowers aquaculturists to implement targeted interventions, such as quarantine measures or treatment strategies, to avert disease outbreaks and minimize economic losses.

Moreover, the development of monoclonal antibodies against PirB paves the way for future research and applications. These antibodies can be harnessed in various diagnostic assays, including ELISA and dot blotting, to enhance the detection sensitivity and specificity of AHPND-causing *Vibrio*. Additionally, the monoclonal antibodies can serve as valuable tools for investigating the pathogenesis of AHPND and exploring potential therapeutic interventions to combat this disease.

In sum, the successful development of monoclonal antibodies against PirB and the establishment of a colloidal gold immunochromatographic assay for the detection of AHPND-causing *Vibrio* constitute significant contributions to the field of aquaculture disease management. These advancements furnish valuable tools for the early and accurate detection of AHPND-causing *Vibrio*, thereby bolstering sustainable shrimp aquaculture practices and ensuring the health and productivity of shrimp populations. Future research endeavors can leverage these findings to further enhance disease surveillance, control measures, and therapeutic interventions in aquaculture settings.

## 5. Conclusions

We successfully developed monoclonal antibodies against PirB and established a colloidal gold immunochromatographic assay for the detection of AHPND-causing *Vibrio*. These advancements provide valuable tools for the early and accurate detection of AHPND-causing *Vibrio*, supporting sustainable shrimp aquaculture practices.

## Figures and Tables

**Table 1:** Bacterial strains and the plasmid used in this study.

Strains and Plasmid	Source/Reference	AHPND-Causing Strain	Culture Medium	Growing Temperature
*V. parahaemolyticus* 20130629002S01 (*Vp*2S01)	PMID: 29051747	+	2216E broth	28 °C
*Vp*2S01::*cat*	PMID: 31231618	+	2216E broth	28 °C
*V. campbellii* LMB29 (*Vc*LMB29)	PMID: 29109705	−	2216E broth	28 °C
*Vc*LMB29-pVPGX1	PMID: 31231618	+	2216E broth	28 °C
*V. campbellii* 20130629003S01 (*Vc*3S01)	PMID: 28050022	+	2216E broth	28 °C
*V. owensii* X170302	Isolated from AHPND water sample in Ruian, Wenzhou, China	+	2216E broth	28 °C
*V. owensii* 20220331001-6	Isolated from AHPND water sample in Ruian, Wenzhou, China	−	2216E broth	28 °C
*V. parahaemolyticus* 20211214002-3	Isolation of AHPND-infected samples in toxicity challenge	−	2216E broth	28 °C
*V. campbellii* 20230718001-5	Isolated from *Penaeus vannamei* sample in Zhejiang, China	+	2216E broth	28 °C
*Alteromonas macleodii* 20170907027	Isolated from *P. vannamei* sample in Zhejiang, China	−	LB broth	37 °C
*Edwardsiella tarda* 20230524001	Lab collection	−	LB broth	37 °C
*Escherichia coli* BL21	Lab collection	/	LB broth	37 °C
*E. coli* expression vector pET-30a(+)	Lab collection	/	/	/

+: AHPND-causing strain; −: non-AHPND-causing strain; /: irrelevant information.

**Table 2:** Indirect ELISA assay results for monoclonal antibody titers.

Dilution Ratio	Mouse #1	Mouse #2	Mouse #3	Mouse #4
200	1.720	1.701	1.631	1.387
400	1.629	1.599	1.498	1.234
800	1.555	1.438	1.310	1.157
1600	1.478	1.359	1.366	1.193
3200	1.359	1.257	1.305	1.136
6400	1.283	1.183	1.064	1.042
12,800	1.126	1.009	0.801	0.810
25,600	0.836	0.716	0.677	0.671
51,200	0.583	0.439	0.456	0.563
102,400	0.390	0.307	0.277	0.388
Blank control	0.023	0.021	0.018	0.001
Negative control	0.068	0.062	0.048	0.028

## Data Availability

The data are contained within the article.

## Supplementary Materials

The following supporting information can be downloaded at https://www.mdpi.com/article/10.3390/ani14111600/s1, Table S1. The detail of the test line analysis; Figure S1: PCR analysis of *pirB* and AP1 gene in strains; Figure S2. The band absorption of different test lines.
