# Supplementary material for: Development of Monoclonal Antibody against PirB and Establishment of a Colloidal Gold Immunochromatographic Assay for the Rapid Detection of AHPND-Causing Vibrio
Source: Animals (Basel). 2024 May 29;14(11):1600. doi: 10.3390/ani14111600 (PMC11171346; doi:10.3390/ani14111600)
Supplement: Supplementary file 1 [file animals-14-01600-s001.zip › Figure S1.pdf]

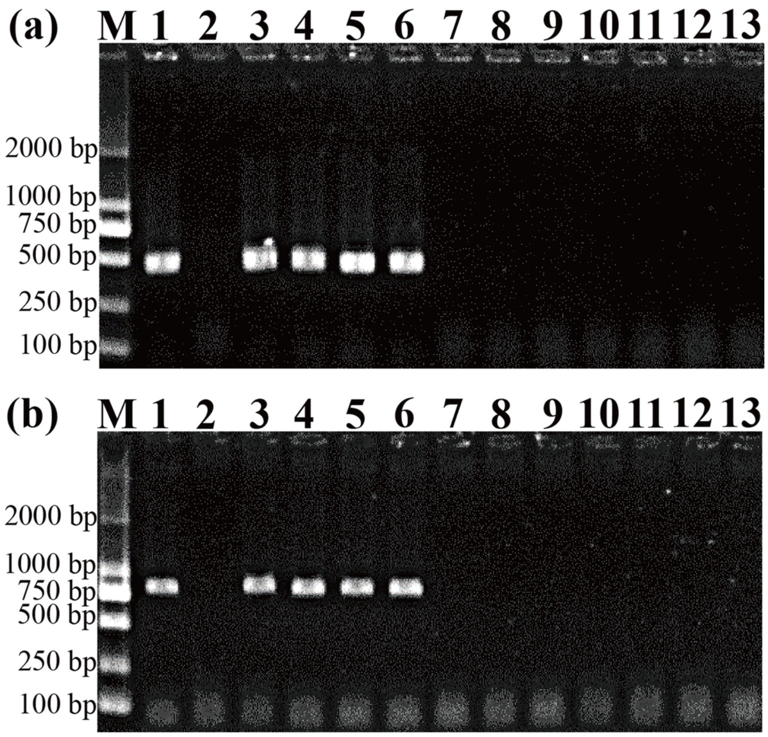

Figure S1. PCR analysis of *pirB* and AP1 gene in strains. (a) Results of *pirB* gene. (B) Results of AP1 gene. M: Marker DL 2000; 1: Postive control (*Vp*2S01::*cat*); 2: Negative control (*Vc*LMB29); 3: *Vp*2S01; 4: *Vc*3S01; 5: X170302; 6: 20230718001-5; 7: *Vc*LMB29; 8: 20211214002-3; 9: 20220331001-6; 10: 20170907027; 11: 20230524001; 12-13: blank control.
