# Supplementary material for: Development of Monoclonal Antibody against PirB and Establishment of a Colloidal Gold Immunochromatographic Assay for the Rapid Detection of AHPND-Causing Vibrio
Source: Animals (Basel). 2024 May 29;14(11):1600. doi: 10.3390/ani14111600 (PMC11171346; doi:10.3390/ani14111600)
Supplement: Supplementary file 1 [file animals-14-01600-s001.zip › Table S1.pdf]

Table S1. The detail of the test Line analysis.

Hours	AHPND	Strains	Strip areas	G	255/G	RA	A
6 hpi	Negative	*Vc*LMB29	Test line	197.2	1.293	0.112	0.001
			Background	197.5	1.291	0.111	
6 hpi	Positive	*Vc*LMB29-pVPGX1	Test line	229.2	1.112	0.046	0.012
			Background	235.5	1.083	0.035	
6 hpi	Positive	*Vp*2S01	Test line	210.5	1.211	0.083	0.013
			Background	217.1	1.174	0.070	
24 hpi	Negative	*Vc*LMB29	Test line	230.7	1.105	0.043	0.001
			Background	231.4	1.102	0.042	
24 hpi	Positive	*Vc*LMB29-pVPGX1	Test line	216.7	1.177	0.071	0.034
			Background	234.1	1.089	0.037	
24 hpi	Positive	*Vp*2S01	Test line	236.8	1.077	0.032	0.015
			Background	245.4	1.039	0.017	
48 hpi	Negative	*Vc*LMB29	Test line	230.2	1.108	0.044	0.000
			Background	230.4	1.107	0.044	
48 hpi	Positive	*Vc*LMB29-pVPGX1	Test line	212.0	1.203	0.080	0.034
			Background	229.4	1.112	0.046	
48 hpi	Positive	*Vp*2S01	Test line	236.3	1.079	0.033	0.010
			Background	242.0	1.054	0.023	

hpi: hours post infection; Positive: AHPND-causing *Vibrio;* Negative: non-AHPND-causing *Vibrio*. The mean gray values (G) at the test line and the background adjacent to the test line were digitalized using ImageJ software. The rough absorbance (RA) was calculated with the formula RA=lg(255/G). The net absorption (A) of the test line resulted from the difference between the RA of the test line and the background $A = RA_{test} - RA_{background}$.
