# Supplementary figures and images for: Development of Monoclonal Antibody against PirB and Establishment of a Colloidal Gold Immunochromatographic Assay for the Rapid Detection of AHPND-Causing Vibrio
Source: Animals (Basel). 2024 May 29;14(11):1600. doi: 10.3390/ani14111600 (PMC11171346; doi:10.3390/ani14111600)

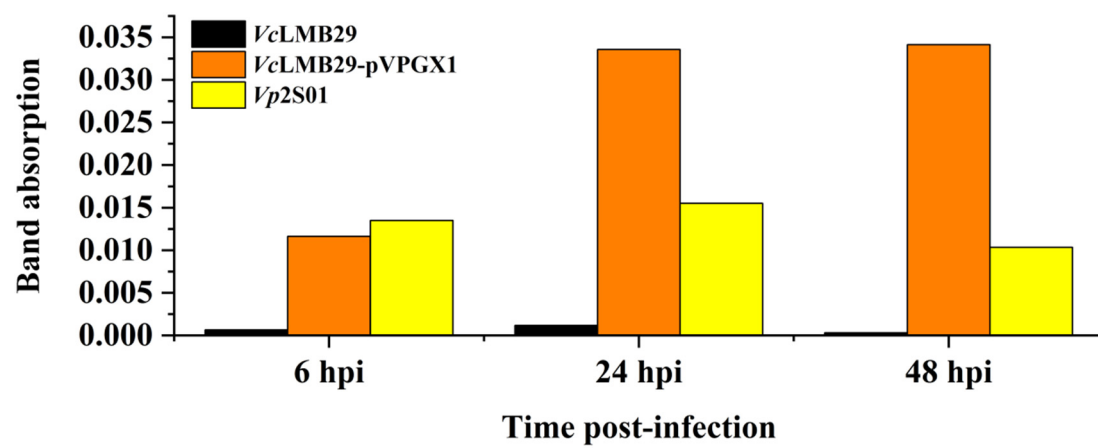

Figure S2. The band absorption of different test lines.

Supplement: Supplementary file 1 [file animals-14-01600-s001.zip › Figure S2.pdf]
